# Proteomics technologies for cancer liquid biopsies

**DOI:** 10.1186/s12943-022-01526-8

**Published:** 2022-02-15

**Authors:** Zhiyong Ding, Nan Wang, Ning Ji, Zhe-Sheng Chen

**Affiliations:** 1 Mills Institute for Personalized Cancer Care, Fynn Biotechnologies Ltd., Gangxing 3rd Rd, High-Tech and Innovation Zone, Bldg. 2, Rm. 2201, Jinan City, Shandong Province 250101 P. R. China; 2 Department of Pharmaceutical Sciences, College of Pharmacy and Health Sciences, Institute for Biotechnology, St. John’s University, 8000 Utopia Parkway, Queens, New York, 11439 USA; 3 Tianjin Medical University Cancer Institute and Hospital, National Clinical Research Center for Cancer, Tianjin’s Clinical Research Center for Cancer, Key Laboratory of Cancer Prevention and Therapy, Tianjin, 300060 China

**Keywords:** Proteomics, Cancer liquid biopsy, Aptamer, Proximity extension assay (PEA), Reverse phase protein arrays (RPPA), Mass spectrometry (MS), Antibody arrays

## Abstract

Alterations in DNAs could not reveal what happened in proteins. The accumulated alterations of DNAs would change the manifestation of proteins. Therefore, as is the case in cancer liquid biopsies, deep proteome profiling will likely provide invaluable and clinically relevant information in real-time throughout all stages of cancer progression. However, due to the great complexity of proteomes in liquid biopsy samples and the limitations of proteomic technologies compared to high-plex sequencing technologies, proteomic discoveries have yet lagged behind their counterpart, genomic technologies. Therefore, novel protein technologies are in urgent demand to fulfill the goals set out for biomarker discovery in cancer liquid biopsies.

Notably, conventional and innovative technologies are being rapidly developed for proteomic analysis in cancer liquid biopsies. These advances have greatly facilitated early detection, diagnosis, prognosis, and monitoring of cancer evolution, adapted or adopted in response to therapeutic interventions. In this paper, we review the high-plex proteomics technologies that are capable of measuring at least hundreds of proteins simultaneously from liquid biopsy samples, ranging from traditional technologies based on mass spectrometry (MS) and antibody/antigen arrays to innovative technologies based on aptamer, proximity extension assay (PEA), and reverse phase protein arrays (RPPA).

## Introduction to proteomics in cancer liquid biopsy

Cancer liquid biopsy has a number of advantages over the traditional tissue biopsy, such as 1) noninvasive or minimally invasive nature of the procedure, which markedly lowers the risk and the cost of the biopsy procedures; 2) providing the systemic and homogenous profiles of all tumor lesions in the human body and overcoming the drawbacks in tissue biopsy caused by intra- or inter-tumoral heterogeneity; and 3) sampling as needed to monitor real-time changes across different stages of cancer evolution. Therefore, liquid biopsy holds the central promise in every aspect of precision medicine and management of cancers, including cancer screening for early detection, diagnostics, prognostics, monitoring patient responses to therapies, and relapses in real time [1, 2]. Liquid biopsy employs minimally invasive procedures to obtain samples for detection. The current widely-used body fluids for liquid biopsies include blood and urine. Theoretically, any other fluid that circulates in or associates with the human body is applicable, including lymphatic fluid, cerebrospinal fluid (CSF), bone marrow, ascites, pleural effusion, cervical fluid, seminal fluid, saliva, sputum, sweat, and stool [3–6].

Biologically, detectable targets in liquid biopsy fall into two categories. One is the cell-free or subcellular structure-free large or small molecules in the body liquid, and those include all primary building blocks of the human body, such as proteins, nucleic acids, lipids, carbohydrates, and other small metabolites and metal ions. The other includes targets with cellular or subcellular structures, including single or clustered circulating tumor cells (CTC), circulating cancer-associated fibroblasts (CAF), immune cells, tumor-educated platelets (TEP) [7], extracellular vesicles (EV), circulating mitochondria [8], and other potential cellular compartments.

Since the 1950s, even before DNA technologies were established, the early concept of cancer liquid biopsy was to examine protein biomarkers from the blood [9]. Over a hundred protein biomarkers were developed for clinical diagnosis in the past decades, with many approved by the Food and Drug Administration (FDA) of the United States. Most comprehensively adopted protein biomarkers, i.e., prostate-specific antigen (PSA), carbohydrate antigen 125 (CA 125), and carbohydrate antigen 19-9 (CA19-9) have been used for cancer diagnosis, monitoring therapeutic responses, or disease recurrence evaluation of prostate, ovarian, and pancreatic cancers, respectively. Nevertheless, the use of most protein biomarkers for early detection and diagnostics still faces an undisputable dilemma due to insufficient specificities and/or sensitivities [10]. Detection of the single or few protein biomarkers in early cancer liquid biopsies relies predominantly on traditional antibody-based approaches, and those include enzyme-linked immunosorbent assays (ELISA), chemiluminescence immunoassays (CLIA), immunohistochemistry (IHC), or liquid-bead immunoassays, which are generalized methodologies in research and clinical practice. However, those approaches suffer from bottlenecks making them unsuitable for high-plex proteomic profiling [11].

The modern concept of cancer liquid biopsy began with the discovery and detection of CTCs and circulating tumor-derived DNA (ctDNA) [12]. In keeping pace with the expansion of research fields, a variety of highly sensitive and specific technologies have been rapidly developed based on multiplex PCR (mPCR) or next-generation sequencing (NGS), facilitating large scale detection of genetic alterations in circulating nucleic acids, such as gene mutations, fusions, deletions, amplifications, translocations, epigenetic changes, and DNA fragmentomics of ctDNA in liquid biopsy studies [1, 13]. Theoretically, technical robustness allows a single DNA or RNA molecule to be detected from reasonable amounts of a standard biological sample. More recently, NGS-based ctDNA or RNA detection methods are making influential changes in modern cancer liquid biopsy due to its ever-increasing sensitivity and specificity.

Notwithstanding the effort made towards nucleic acid-based strategies, the importance of proteomic-based profiling in cancer liquid biopsies never diminishes. Since proteins are the direct executors of most cellular functions and the direct drug targets in most current cancer therapies, high dimensional proteomic data are likely to provide unprecedented insights to aid novel biomarker identification and clinical implementation. Protein profiles from liquid biopsy samples also likely reveal more organ-specific information than DNA or even RNA, which helps to identify tumor origin. In a similar scenario to the DNA/RNA, applying novel protein biomarkers independently or in conjunction with nucleic acids significantly improved diagnostic accuracy [14].

To accomplish this goal, researchers have been striving to upscale the dimensionality of protein biomarker profiling in the perspective of either covering the entire proteome or deep diving into the post-translational modifications (PTM). From a proteogenomic perspective, quantitative measurement of the proteome is more challenging technically and theoretically than assessing the genome. Firstly, as compared to a total of 22,000 to 25,000 protein-translatable genes within the human genome, the proteome is expected to encompass over one million different proteoforms through various epigenetic regulations, different RNA splicing, and PTM. Moreover, the dynamic range of proteins spans up to 12 logs of magnitude in cells or body fluids [15]. Lastly, the proteome is in constant and rapid changes in protein abundances and/or modifications, responding to all kinds of stimuli. While you cannot measure the same proteome twice, in contrast, the genome is relatively stable with slow constant changes. These challenges are why proteomics usually lag behind genomics in many applications.

**Fig. 1:**
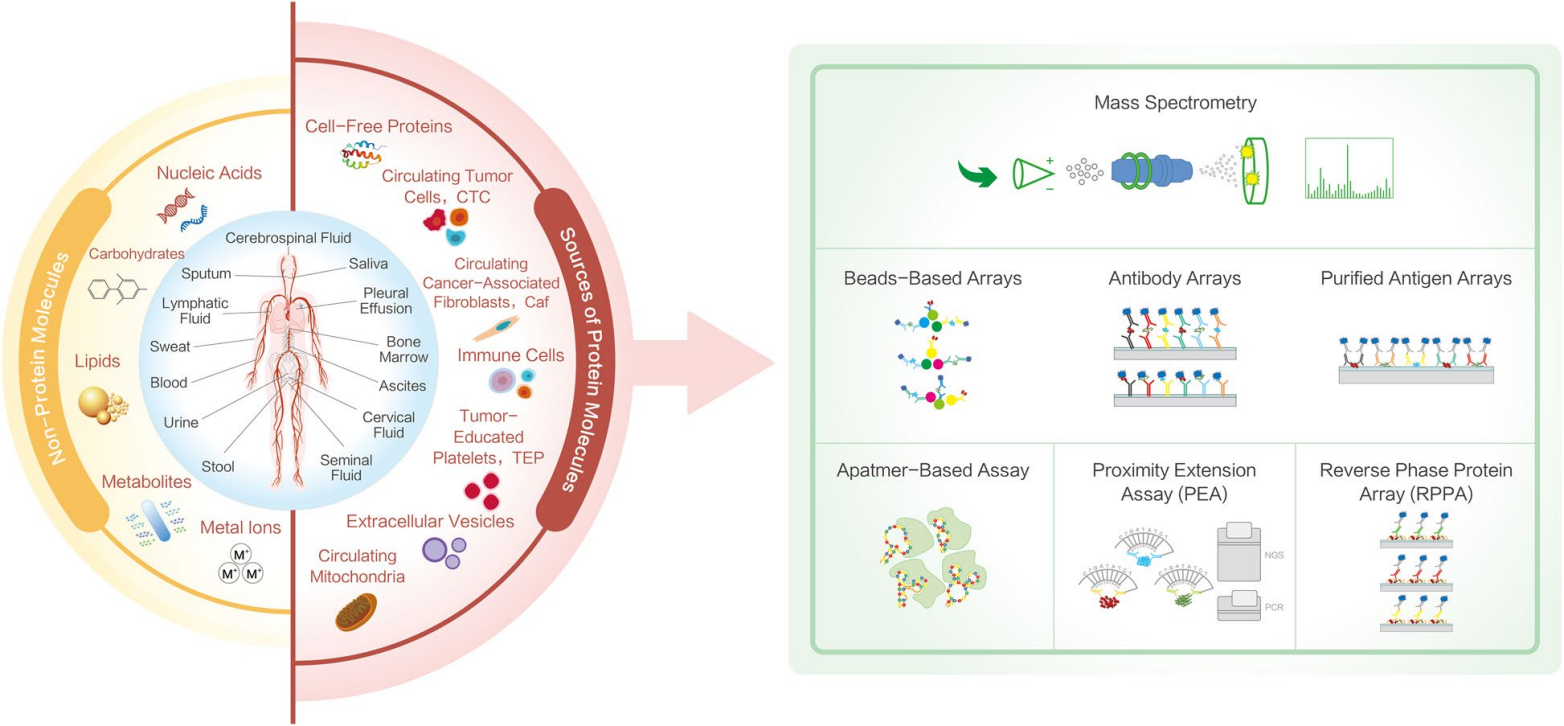
Overview of proteomics technologies in cancer liquid biopsies. The inner ring (blue) in the left panel describes the origins of all types of body fluids (Blood, urine, stool, seminal fluid, cervical fluid, ascites, bone marrow, pleural effusion, saliva, CSF, sputum, lymphatic fluid, and sweat). The outer ring is two-colored denoting non-protein (yellow) and sources of protein molecules (red) that are potential biomarkers of interest, the latter of which is further connected with discovery proteomics technologies with demographic principles (right green panel). Those technologies include mass spectrometry, reverse phase protein array, antibody arrays/antigen arrays/beads arrays, proximity extension assay, and aptamer assay and are discussed in this review

Despite the challenges, the irreplaceable values and clinical demands of novel proteomic biomarkers in cancer liquid biopsies bring ever-growing excitement in the research communities to revolutionize technologies to understand the proteome better. In this respect, breakthroughs have been made in recent years with either advancement in existing technologies or the advent of innovative methodologies. In this review, we focus on both mainstream and groundbreaking high-plex proteomics technologies, each with the analytical scope of characterizing hundreds to thousands of protein targets simultaneously from a liquid biopsy sample and discuss their advantages, shortcomings, and potential applications in cancer liquid biopsies (Fig. 1).

## High-plex proteomics technologies applied in cancer liquid biopsy

### Mass spectrometry (MS)

MS-based proteomics has long been a powerful tool for cancer biomarker profiling in the context of various body fluids, where the predominant focuses are based on serum/plasma and urine. In liquid biopsy profiling, with the technical and methodological advances, modern MS mainly adopts purpose-designed sample preparation together with liquid chromatography (LC) prior to peptide ionization and tandem MS scans [16]. A hypothetical LC-MS setup involves sample digestion followed by peptide titration. Diluted peptide fractions are ionized and characterized in a mass analyzer. Due to biofluids’ complex characteristics, especially blood, endeavors have been taken to increase the number of proteins to be characterized in precision [17, 18]. Those include optimizing preparation workflow (immunodepletion/filter-aided sample preparation [FASP], MStern blotting, suspension trapping [S-trap]), development of quantification techniques (isobaric labeling/label-free), changes in the MS scanning modes (data-dependent acquisition [DDA], data-independent acquisition [DIA]), and instrumentation advancements (high-field asymmetric ion mobility spectrometry [FAIMS]/trapped ion mobility spectrometry [TIMS]) [16, 19].

A key advantage of MS for cancer liquid biopsy is that it allows non-hypothesis-driven proteomic research (total proteins and modified forms), making it a preferred approach at the early biomarker discovery stage. Currently, for clinical proteomic profiling, a few hundred to over a thousand proteins can be characterized in an untargeted MS run in serum or plasma, whereas several thousand targets can be achieved simultaneously in urine-based MS profiling owning to its much less complex protein composition [16, 17]. In blood-based proteomics, the critical task is to suppress the noise or false discovery rate due to the enormous dynamic range of blood protein content as well as pre-analytical variations [18, 20]. However, MS-based liquid biopsies have been employed in multiple cancers, including lung, breast, colorectal, ovarian, gastric, pancreatic, prostate, cervical, lymphoma, and so forth [18]. Most of the studies span from cancer screening to diagnosis and prognosis for both local and advanced diseases. By combining an ultra-depletion method with four types of fractionation method together with label-free Sequential Window Acquisition of all Theoretical Mass Spectra (SWATH-MS) DIA, researchers explored colorectal cancer (CRC) serological diagnostic markers on a cohort of 100 plasma samples from healthy and stage I-IV patients, and identified 513 plasma proteins within which seven were further validated by Western blotting and/or ELISA [21]. Furthermore, a 5-protein signature had accurate predictive power to discern early and late-stage CRC [21]. In a longitudinal study, paired serum samples from 6 advanced gastric cancer patients were used pre- and post-operation for LC-MS/MS profiling, and SOX3 was identified as a potential prognostic marker [22]. Besides, urine-based proteomics is another avenue to explore due to its much higher target plexity and is particularly suited for urological cancers. Work from a two-step biomarker profiling discovered a 34-marker protein panel which was further validated in an independent cohort [23]. MS-based cancer liquid biopsies were extensively reviewed in the literature [16, 19].

Of importance, one successfully implemented protein biomarker panel for early-stage ovarian cancer has already become available in clinical practice (OVERA) and fundamental discovery and multi-center cross-validation using surface-enhanced laser desorption/ionization (SELDI)-based MS method, which made significant impact in this field [24, 25]. However, fundamental hurdles in MS-based methods for translational research still need to be overcome and a streamlined development work flow is indispensable for successful clinical implementation [18]. A triangular strategy starting with de novo MS discovery that can be transferred to medium- or low-plex targeted proteomic platforms for downstream verification seems to be a widely-adopted approach; however, a rectangular strategy using deep-discovery MS, targeted MS (single reaction monitoring [SRM] or multiple reaction monitoring [MRM]), and other high-resolution MS methods throughout the biomarker profiling phases is also proposed [19]. All those together would ensure the discovery of true tumor-associated proteins (TAP) translatable to clinical settings [26].

### Antibody/antigen arrays

For simplicity purposes, we put planar and bead antibody arrays together with proteome arrays in this section due to their shared biochemical and analytical properties. Though bead-based arrays and sandwich ELISA-based planar array are widely used, their analytical scope is mainly confined within medium/low-plex proteomics profiling and thus will not be discussed here [27]. As one of the early developed targeted proteomic tools, antibody arrays have been applied in various contexts for cancer proteomics studies [28]. A typical technical setup involves immobilizing specific antibodies onto modified planar substrates via covalent binding, affinity binding, or physical entrapment [29, 30]. In high-plex (typically several hundred targets) profiling, samples are preferably labeled with fluorescent, chemiluminescent, or oligo-coupled tags to allow different signal amplification and detection. This method can practically characterize over a thousand proteins or modified proteoforms with minimal immunogenic cross-relativity induced from antibody reaction mixtures.

Antibody arrays have ultraperformance for knowledge-based biological interrogation that can overcome sensitivity issues associated with untargeted proteomic techniques. Antibody arrays are particularly useful for serological profiling as most of the TAP are low abundant cellular efflux such as hormones, cytokines, chemokines, intracellular signaling molecules and post-translational modifications [31]. Antibody array has been applied in bladder cancer in seeking diagnosis signatures [32]. Its applications in prostate cancer, ovarian cancer, CRC, and others have also been indicated [28, 33]. Nevertheless, its suboptimal quantification due to narrow dynamic ranges and signal saturation, sample labeling prerequisite, and inter-assay variation make it a small methodological niche for biofluid-based proteomic profiling.

Antigen arrays, also named functional protein arrays, form another high-throughput discovery proteomics field, and its application in biofluid-based research has been broadly adopted [34, 35]. Functional protein arrays start with the deposition of ectopically expressed proteins/peptides with a wide range of proteome coverage in species of interest, and these serve as baits to capture analytes of interest within the flowthrough. It can theoretically investigate protein interaction with proteins (protein PTMs), lipids, cells, small molecules, nucleic acids, and antibodies. Serological autoantibodies (AAbs) are a hotspot for cancer biomarker profiling in this aspect [34].

At present, the most comprehensive human proteome array reaches over 81% proteome coverage (21,000 protein forms), making it a robust tool to obtain a panoramic landscape of blood proteomics [34, 36]. A panel of lung cancer early diagnostic AAbs (against p53, H-Ras, and ETHE1) were identified using high-plex protein arrays [37]. A longitudinal study was also conducted to identify therapy-associated AAb signatures in lung cancer patients [38]. As a target-focused validation tool, it was used in part during the development of the clinically approved lung cancer early detection AAb panel (EarlyCDT), whereby some specific antigen arrays were used for expanded cohort studies such as NY-ESO-1 [39, 40]. Many other serological AAb markers have also been explored in ovarian, gastric, bladder, prostate, and breast cancers [34]. Though excellent as an exploratory tool in serological AAb profiling, the scalability, reproducibility, inter-assay variation, and costs of antibody/antigen arrays remain the pitfalls when designing the entire study pipeline.

### Aptamer-based assays

Aptamers are short single-stranded DNA or RNA, or peptides that, upon folding into specific tertiary structures, bind to cognate protein targets in native states with high affinity and specificity [41–43]. The current approach, in the case of slow off-rate modified aptamers (SOMA) scan assay, incorporates binding molecules (SOMAmers) attached to photocleavable linkers and fluorescent labels, and those nucleic acid structures are then used to capture proteins of interest followed by biotin-mediated purification, oligo release via ultraviolet (UV)-based cleavage and tagging of bound proteins with biotin. The protein-bound SOMAmers are then eluted off for characterization and quantification via conventional DNA hybridization techniques, reflecting the protein abundance within the system [44]. Aptamers are more advantageous in their higher affinity and specificity than antibodies, and they can be readily synthesized and selected in vitro with low batch-to-batch variation, providing a cost-efficient way to scale up its multiplexity [45]. The ultra-high specificity of the aptamer was demonstrated by a study showing an RNA aptamer with a 10,000-fold higher affinity for theophylline than caffeine, two molecules different in only one methyl group [46]. Improvements have also been made to expand the analytical diversity of aptamers by introducing chemically modified nucleotides to mimic amino acid side chains [47]. It is conceivable that the aptamers containing modified side chains could have many more different structures that improve their binding properties. The specificity of aptamers was also significantly improved by a modified version (SOMAmers) to allow non-specific bindings being disrupted by an anionic competitor while maintaining on-rates for true targets. This enables a high-throughput ultra-plex screening approach with more than 7000 proteins to be profiled in parallel [47].

In cancer liquid biopsy, a recent stool-based profiling, a 1317 protein-based aptamer screening revealed multiple protein signatures to identify CRC patients from healthy controls or adenoma [4]. Of more clinically relevant, an early aptamer-based study measured 813 proteins in 1326 non-small cell lung cancer (NSCLC) serum samples and controls and identified multiple protein biomarkers potentially as early detection of NSCLC [48]. This work directly led to the consequent validation and successful implementation of a 7-protein biomarker panel in clinical settings (AptoDetect-Lung) [49].

Regardless of the number of protein-specific aptamers that have now reached over 7000 for commercial assay services, one limitation is the difficulty of developing high-quality aptamers for novel targets. Aptamers available to the research communities are still limited compared with antibodies. In addition, its exploration of PTM biomarkers is yet preliminary, although some PTM-oriented aptamer development such as phosphor-specific aptamer sporadically existed [50].

### Proximity extension assay (PEA)

PEA takes advantage of the concept adopted in conventional sandwich ELISA and the readiness of highly specific and sensitive DNA-readout methodologies (quantitative PCR/NGS), creating a smartly designed proteomic detection technology particularly suited for liquid biopsy-based discovery [51]. Its broad dynamic range (scan 10 logs) and minimal sample requirement make it an excellent tool for serological profiling. In PEA, multiple antibody pairs for proteins of interest are pooled. Each antibody in a pair is labeled with complementary DNA oligo sequences to allow high-fidelity discriminative hybridization, a process that only happens when true antibody pairs are brought into proximity by binding to the target proteins [52]. The resultant double-stranded DNA sequences are PCR-amplified. Real-time PCR (in a medium-to-low plex manner) or NGS (in a high-plex manner) is used as the readout to measure the relative concentration of the target proteins [5]. The most advanced PEA assay has a standard measurement coverage of 3072 (commercialized by Olink) targets, and by avoiding the cross-reactivity issue raised in multiplexed immunoassays, and the analytical scope can further grow in principle [52].

As a robust serological discovery tool, PEA was first applied to identify prognostic biomarkers from blood in CRC [53], and promising plasma protein biomarker panels were identified, validating the strength and potential of this technique. It was also extended to other cancer types for serological proteomic profiling, including cervical, ovarian, prostate, lung, and hematopoietic cancer for early detection, companion diagnostics, and disease monitoring [54–58]. Owing to its high sensitivity in targeted detection, PEA has outperformed LC-MS methods, presenting wider dynamic ranges with high accuracy and reproducibility within the pg/ml ranges [59]. With this aid, large-scale biomarker profiling in gynecologic tumors based on 441 PEA targets showed a panel of 27-protein biomarkers to distinguish benign tumors and high-grade ovarian cancer with a sensitivity of 0.88 and specificity of 0.92 (AUC = 0.92), and its diagnostic performance was significantly better than conventional CA125 and human epididymis protein 4 (HE4) biomarkers [56]. The panel was also validated for population screening with a sensitivity of 0.85 and a specificity of 0.92 (AUC = 0.89) [56]. The finding was further strengthened based on a 593 PEA protein profiling in larger sample cohorts, confirming an 11 biomarker panel for ovarian cancer diagnostics and population screening [60]. Notably, applications in cellular lysates and single cells started to emerge, opening new avenues for integrative multi-omics profiling [61, 62].

Nevertheless, the trade-off in high-plex discovery PEA (in the case of more than 96-plex) is the library preparation and NGS requirement, an analytical factor to consider due to biases and intra−/inter-experimental variations when high sample size throughput is in place. Therefore, quantitative detection of over a thousand protein targets still needs real-world validation in the near future.

### Reverse phase protein arrays (RPPA)

RPPA emerged two decades ago and was consequently developed into a high-throughput, high-content targeted proteomics technology superior to tissue-based profiling, especially for tracking proteins and PTM within signaling networks [63]. RPPA is an open-source technology that can be assembled in various ways [64]. In a typical RPPA setup, fully denatured protein lysates are immobilized onto solid substrates, usually with dilution series, and this process can be repeated to allow any number of targets to be interrogated (currently up to 500 targets). Sample-containing slides are probed with highly specific antibodies pre-validated for RPPA application, and quantitative signals are captured through either colorimetric amplification or fluorescence detection. RPPA is super robust in parallel to large sample profiling due to its nature of quantifying all samples in one experimental run, which usually ranges from a few hundreds to over a thousand samples [63, 65, 66]. Depending on antibody availability, RPPA can be broadly used for proteins, protein isoforms, and PTM, including phosphorylation, methylation, and acetylation analysis [67, 68]. Additionally, its minimal pre-experimental process in a complete denaturing condition increases detection and quantification accuracy, allowing subtle fluctuations to be captured in biological systems. RPPA is the most systemically adopted technology for large-scale patient profiling in solid tumors and leukemia. This is featured by its extensive application in The Cancer Genome Atlas (TCGA) project, and the public data sets can be accessed via The Cancer Proteome Atlas portal (TCPA, http://tcpaportal.org) [69].

Due to its wide application advantages and its ability to track intra-cellular proteins, RPPA has been well adopted in blood cancer and other liquid biopsies [65, 70, 71]. It was previously compared to ELISA in examining CA19-9 in serum and plasma, and showed increased sensitivity [72]. In a lung cancer study, RPPA was utilized to profile more than 370 serum samples for candidate biomarkers, an approach crucial for biomarker validation [73]. The super-high throughput also allowed for parallel profiling of over 12,000 clinical blood samples in one experiment [71]. More significantly, RPPA can be employed as a robust validation tool of protein biomarker validation due to its minimal inter-assay variation and has been successfully applied in previous novel biomarker validation in lung cancer [74].

Tumor-derived EVs, such as exosomes that may potentially harbor oncoproteins in situ, have become another hot spot for RPPA application in cancer liquid biopsy [75]. A preliminary study showed a size-exclusion chromatography-based EV purification workflow compatible with downstream RPPA analysis of 276 cellular proteins, finding seven protein biomarkers to distinguish breast cancer patients from healthy people with both predictive and prognostics power [76]. A recent RPPA study on EVs from prostate cancer patient sera also validated protein biomarkers with potential prognostic and predictive values [77].

Methodology-wise, RPPA requires sophisticated experimental workflow, including key steps such as array printing, multiple steps of immunostaining and signal amplification, high-resolution data readout, and homebrewed data compiling and analysis [64]. Besides, the prolonged experimental process, especially when higher plex discovery proteomics is needed, may slow the turnaround time. The validation of RPPA-usable antibodies is another bottleneck to consider due to the antigen-down immunoreaction format.

## Conclusions

### Summary

We briefly reviewed the current progress of the existing high-plex proteomic technologies under the context of cancer liquid biopsy and summarized the advantages and disadvantages of this application (Table 1). From a clinical translational point of view, since the repertoire of tumor liquid biopsy-based proteomic biomarkers in current use is only less than 40, most are for diagnostic purposes (https://www.cancer.gov/about-cancer/diagnosis-staging/diagnosis/tumor-markers-list), it poses a paramount need to implement novel protein-based biomarkers under appropriate clinical settings. A marked increase in innovative technologies and conventional platforms to acquire high dimensional data both sample-wise and target-wise are being observed. These technologies also grow exponentially in the field of cancer liquid biopsy.

**Table 1:** Key application features of proteomics technologies for cancer liquid biopsies

	Basic principles	Typical multi-plexity	Typical sample throughput (per assay)	Limit of Detection/ Dynamic range	Readout	De-novo/ targeted	Laboratory procedures	Advantages	Limitations
**Mass Spec (MS)**	Samples are prepared according to specific purposes, followed by digestion, peptide ionization, and tandem MS scans.	~ 6500 (Urine) ~ 1500 (blood)	Up to 16 (TMT labelling)	Picogram/ 4-5 logs	Fragmented peptide sequences and spectrum counting	De-novo/ Targeted	Heavy instrumentation and non-standardized workflows for pre-MS sample preparation Semi-automated	De-novo process suitable for exploratory research	Low throughput, Complex depletion process, limitations to analyze protein PTMs
**Antibody/Antigen Arrays**	Antibodies/Antigens immobilized onto the solid substrates. Targets proteins in samples captured by the antibodies/antigens.	~ 4000 (antibody arrays)/ up ~ 21,000 (antigen arrays)	~ 1-64	Low picogram/ 5 logs	Colorimetric assays or fluorescence	Targeted	Standardized workflows, Semi-automated	Widely adopted approach with flexible experimental design and PTM profiling.	Limit on inter-assay reproducibility and quantification, inter-assay variation, Sample labelling
**Aptamer-based Assay**	Short single-strand DNA or RNA fold into specific tertiary structures with the ability to bind targets with high affinity and specificity.	~ 7000	Up to 1000	Low picogram/ 4-5 logs	DNA microarray/ fluorescence	Targeted	Standardized workflows performed at service centers, Semi-automated	High-plexity	Limit on protein PTMs detection; Dependence on DNA microarray for readout
**Proximity extension assay (PEA)**	Sandwich ELISA labeled with complementary DNA oligos binds to the target allowing the oligos to hybridized.	~ 3000	88	Low picogram to femtogram/ 10 logs	qPCR (medium-plex) or NGS (high-plex)	Targeted	Standardized workflows with Semi-automated	Very little sample required with large dynamic ranges	Dependence on Q-PCR/NGS for readout
**Reverse phase protein arrays (RPPA)**	Protein samples immobilized onto the solid substrates. Targets detected by antibodies with signal amplification.	~ 500	Up to 1056	Femtogram/ 5-6 logs	Colorimetric assays or fluorescence	Targeted	Heavy instrumentation and non-standardized workflows, Semi-automated	Large scale parallel analysis for samples. Suitable for protein PTMs	Relatively long turnaround time

### Future perspectives

One future direction is the development on the technical side of proteomics. Increasing the detection resolution, standardizing workflows, and expanding high-quality antibodies with high sample throughput will leverage the overall detection accuracy, especially at the early discovery stage. This is simply because most organ-specific biomarkers in secretome are present at ultra-low abundance and are yet to be detected. As many of the development only took place in recent years, we foresee an accelerated rate of candidate biomarker expansion in the upcoming years within the field of cancer liquid biopsy.

**Fig. 2:**
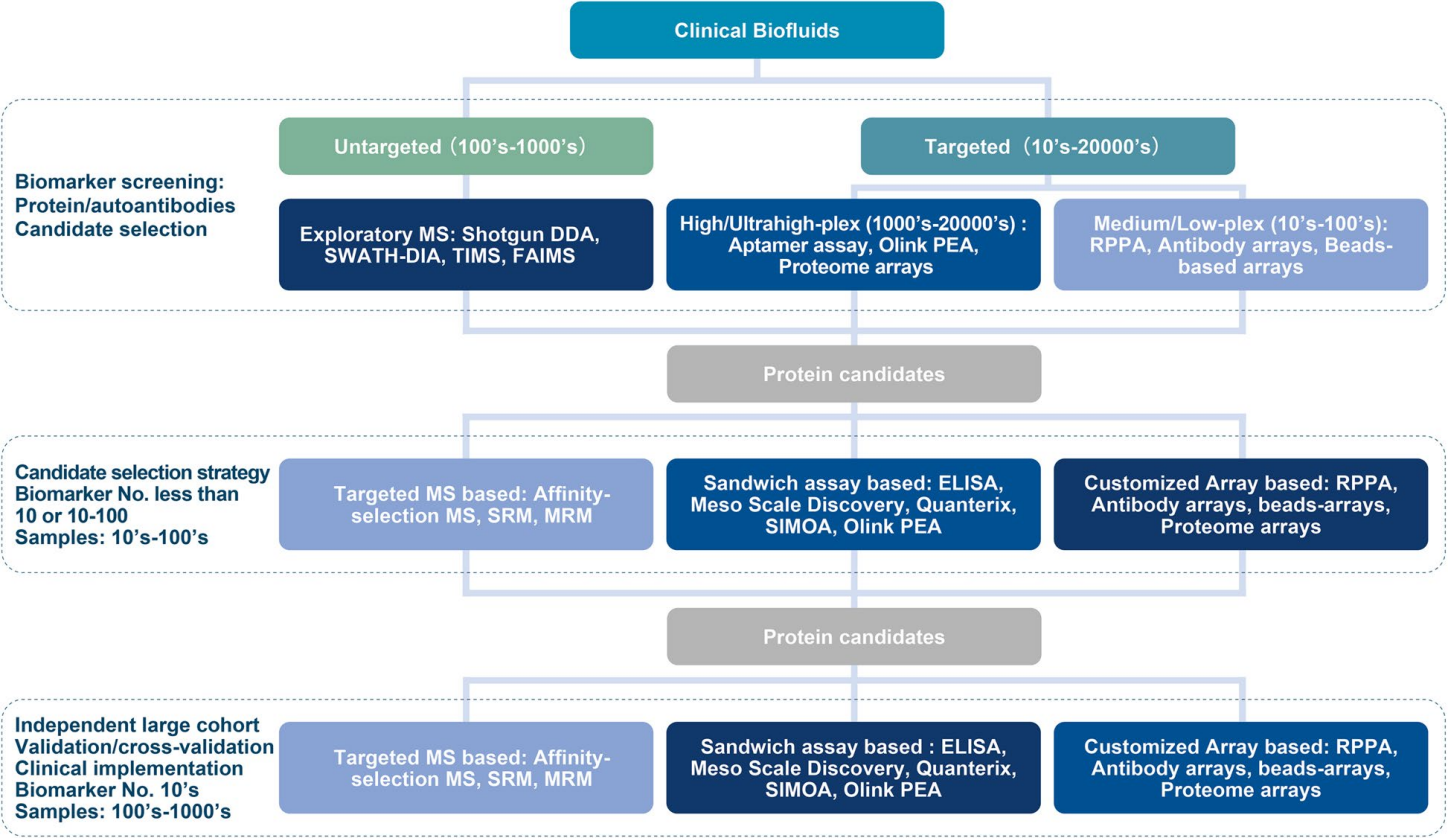
Proteomics-based cancer liquid biopsy for translational medicine. A workflow of clinical biomarker discovery divided into three stages (biomarker screening, candidate selection, and large-scale validation and implementation). Untargeted and targeted routes for biomarker exploratory with their analytical scopes are shown. Intensities of the blue color denote their probable significance at individual stages

Another developmental direction is to establish feasible strategies by joint applications of different proteomics technologies for orthogonally validating biomarker candidates and complementing weaknesses. Given the non-existence of a so-called perfect technology, balancing the advantages and disadvantages between individual technologies throughout the development stages is pivotal [59]. This can be seen in recent studies, where combinations of MS or aptamers with PEA were used for cancer biomarker discovery [78, 79]. We hypothetically proposed a simple pipeline by incorporating all methods mentioned above and a few others, with colored intensity denoting the prevalence of each at discovery, validation, and clinical implementation stages. The tendency may be subject to change in the near future (Fig. 2).

Lastly, single-cell proteomics is also sweeping across all fields of cancer biomarker discovery, and as in the case of liquid biopsy. Evaluating CTC was a hallmark to take the single-cell proteomics onto a new horizon. As some CTC-based assays have already become actionable tests for cancer patients to predict progression-free survival and overall survival, a deep dive into proteomics at individual cell levels will be enchanting to the scientific community [80]. MS has already paved the way in single-cell proteomics with the aid of flow-cytometry cell sorter and high-resolution TIMS-TOF [81]. Surface protein phenotypes and single-cell secretome, particularly in cancer immunotherapy, are both hotspots to search for new biomarkers in liquid biopsy [82, 83]. All these will open up a new treasure box in CTC-based exploratory biomarker profiling.

With various technological advancements in every aspect, proteomic-based biomarker discovery can be fundamentally redefined in the ballpark of cancer liquid biopsy. We look forward to a more streamlined and coherent biomarker profiling workflow with the ultimate application in cancer medicine.

## Data Availability

Not Applicable.

## Abbreviations

PEA: Proximity Extension Assay
RPPA: Reverse Phase Protein Arrays
MS: Mass Spectrometry
CSF: Cerebrospinal fluid
CTC: Circulating tumor cell
CAF: Circulating cancer-associated fibroblast
TEP: Tumor-educated platelet
EV: Extracellular vesicle
FDA: The US Food and Drug Administration
PSA: Prostate-specific antigen
CA125: Carbohydrate antigen 125
CA19-9: Carbohydrate antigen 19-9
ELISA: Enzyme-linked immunosorbent assay
CLIA: Chemiluminescence immunoassay
IHC: Immunohistochemistry
ctDNA: Circulating tumor-derived DNA
mPCR: Multiplex PCR
NGS: Next-generation sequencing
PTM: Post-translational modification
LC: Liquid chromatography
FASP: Filter-aided sample preparation
S-trap: Suspension trapping
DDA: Data-dependent acquisition
DIA: Data-independent acquisition
FAIMS: High-field asymmetric ion mobility spectrometry
TIMS: Trapped ion mobility spectrometry
SWATH-MS: Sequential Window Acquisition of all Theoretical Mass Spectra
SELDI: Surface-enhanced laser desorption/ionization
CRC: Colorectal cancer
SRM: Single reaction monitoring
MRM: Multiple reaction monitoring
TAP: Tumor-associated protein
AAb: Autoantibody
SOMAmer: Slow off-rate modified aptamer
UV: Ultraviolet
NSCLC: Non-small cell lung cancer
HE4: Human epididymis protein 4
TCGA: The Cancer Genome Atlas
TCPA: The Cancer Proteome Atlas

## References

[1] Alix-Panabières C, Pantel K (2021). Liquid biopsy: from discovery to clinical application. Cancer Discov.

[2] Rossi G, Ignatiadis M (2019). Promises and pitfalls of using liquid biopsy for precision medicine. Cancer Res.

[3] Komor MA, Bosch LJ, Coupé VM, Rausch C, Pham TV, Piersma SR (2020). Proteins in stool as biomarkers for non-invasive detection of colorectal adenomas with high risk of progression. J Pathol.

[4] Li H, Vanarsa K, Zhang T, Soomro S, Cicalese PA, Duran V (2021). Comprehensive aptamer-based screen of 1317 proteins uncovers improved stool protein markers of colorectal cancer. J Gastroenterol.

[5] Suhre K, McCarthy MI, Schwenk JM (2021). Genetics meets proteomics: perspectives for large population-based studies. Nat Rev Genet.

[6] Amelio I, Bertolo R, Bove P, Buonomo OC, Candi E, Chiocchi M (2020). Liquid biopsies and cancer omics. Cell Death Discov.

[7] In ‘t Veld SGJG, Wurdinger T. (2019). Tumor-educated platelets. Blood.

[8] Song X, Hu W, Yu H, Wang H, Zhao Y, Korngold R (2020). Existence of circulating mitochondria in human and animal peripheral blood. Int J Mol Sci.

[9] Rifai N, Gillette MA, Carr SA (2006). Protein biomarker discovery and validation: the long and uncertain path to clinical utility. Nat Biotechnol.

[10] Hanash SM, Pitteri SJ, Faca VM (2008). Mining the plasma proteome for cancer biomarkers. Nature.

[11] Landegren U, Hammond M (2021). Cancer diagnostics based on plasma protein biomarkers: hard times but great expectations. Mol Oncol.

[12] Palmirotta R, Lovero D, Cafforio P, Felici C, Mannavola F, Pelle E (2018). Liquid biopsy of cancer: a multimodal diagnostic tool in clinical oncology. Ther Adv Med Oncol.

[13] Ignatiadis M, Sledge GW, Jeffrey SS (2021). Liquid biopsy enters the clinic — implementation issues and future challenges. Nat Rev Clin Oncol.

[14] Cohen JD, Li L, Wang Y, Thoburn C, Afsari B, Danilova L (2018). Detection and localization of surgically resectable cancers with a multi-analyte blood test. Science.

[15] Keshishian H, Burgess MW, Specht H, Wallace L, Clauser KR, Gillette MA (2017). Quantitative, multiplexed workflow for deep analysis of human blood plasma and biomarker discovery by mass spectrometry. Nat Protoc.

[16] Macklin A, Khan S, Kislinger T (2020). Recent advances in mass spectrometry based clinical proteomics: applications to cancer research. Clin Proteomics.

[17] Hu S, Loo JA, Wong DT (2006). Human body fluid proteome analysis. Proteomics.

[18] Bhawal R, Oberg AL, Zhang S, Kohli M (2020). Challenges and opportunities in clinical applications of blood-based proteomics in cancer. Cancers (Basel).

[19] Geyer PE, Holdt LM, Teupser D, Mann M (2017). Revisiting biomarker discovery by plasma proteomics. Mol Syst Biol.

[20] Anderson NL, Polanski M, Pieper R, Gatlin T, Tirumalai RS, Conrads TP (2004). The human plasma proteome: a nonredundant list developed by combination of four separate sources. Mol Cell Proteomics.

[21] Ahn SB, Sharma S, Mohamedali A, Mahboob S, Redmond WJ, Pascovici D (2019). Potential early clinical stage colorectal cancer diagnosis using a proteomics blood test panel. Clin Proteomics.

[22] Shen J, Zhai J, Wu X, Xie G, Shen L (2020). Serum proteome profiling reveals SOX3 as a candidate prognostic marker for gastric cancer. J Cell Mol Med.

[23] Kim Y, Jeon J, Mejia S, Yao CQ, Ignatchenko V, Nyalwidhe JO (2016). Targeted proteomics identifies liquid-biopsy signatures for extracapsular prostate cancer. Nat Commun.

[24] Bast RC, Lu Z, Han CY, Lu KH, Anderson KS, Drescher CW (2020). Biomarkers and strategies for early detection of ovarian Cancer. Cancer Epidemiol Biomark Prev.

[25] Zhang Z, Bast RC, Yu Y, Li J, Sokoll LJ, Rai AJ (2004). Three biomarkers identified from serum proteomic analysis for the detection of early stage ovarian cancer. Cancer Res.

[26] Borrebaeck CA (2017). Precision diagnostics: moving towards protein biomarker signatures of clinical utility in cancer. Nat Rev Cancer.

[27] Krishnan VV, Selvan SR, Parameswaran N, Venkateswaran N, Luciw PA, Venkateswaran KS (2018). Proteomic profiles by multiplex microsphere suspension array. J Immunol Methods.

[28] Kopf E, Zharhary D (2007). Antibody arrays--an emerging tool in cancer proteomics. Int J Biochem Cell Biol.

[29] Haab BB (2005). Antibody arrays in cancer research. Mol Cell Proteomics.

[30] Reslova N, Michna V, Kasny M, Mikel P, Kralik P (2017). xMAP technology: applications in detection of pathogens. Front Microbiol.

[31] Yu X, Schneiderhan-Marra N, Joos TO (2010). Protein microarrays for personalized medicine. Clin Chem.

[32] Sanchez-Carbayo M, Socci ND, Lozano JJ, Haab BB, Cordon-Cardo C (2006). Profiling bladder cancer using targeted antibody arrays. Am J Pathol.

[33] Sanchez-Carbayo M (2006). Antibody arrays: technical considerations and clinical applications in cancer. Clin Chem.

[34] Syu GD, Dunn J, Zhu H (2020). Developments and applications of functional protein microarrays. Mol Cell Proteomics.

[35] Duarte JG, Blackburn JM (2017). Advances in the development of human protein microarrays. Expert Rev Proteomics.

[36] Jeong JS, Jiang L, Albino E, Marrero J, Rho HS, Hu J (2012). Rapid identification of monospecific monoclonal antibodies using a human proteome microarray. Mol Cell Proteomics.

[37] Pan J, Song G, Chen D, Li Y, Liu S, Hu S (2017). Identification of serological biomarkers for early diagnosis of lung cancer using a protein array-based approach. Mol Cell Proteomics.

[38] Li Y, Li CQ, Guo SJ, Guo W, Jiang HW, Li HC (2020). Longitudinal serum autoantibody repertoire profiling identifies surgery-associated biomarkers in lung adenocarcinoma. EBioMedicine.

[39] Shan Q, Lou X, Xiao T, Zhang J, Sun H, Gao Y (2013). A cancer/testis antigen microarray to screen autoantibody biomarkers of non-small cell lung cancer. Cancer Lett.

[40] Lam S, Boyle P, Healey GF, Maddison P, Peek L, Murray A (2011). EarlyCDT-lung: an immunobiomarker test as an aid to early detection of lung cancer. Cancer Prev Res (Phila).

[41] Huang J, Chen X, Fu X, Li Z, Huang Y, Liang C. Advances in aptamer-based biomarker discovery. Front Cell Dev Biol. 2021;9(571):1–11. 10.3389/fcell.2021.659760 PMC8007916 33796540

[42] Tuerk C, Gold L (1990). Systematic evolution of ligands by exponential enrichment: RNA ligands to bacteriophage T4 DNA polymerase. Science.

[43] Ellington AD, Szostak JW (1990). In vitro selection of RNA molecules that bind specific ligands. Nature.

[44] Brody EN, Gold L, Lawn RM, Walker JJ, Zichi D (2010). High-content affinity-based proteomics: unlocking protein biomarker discovery. Expert Rev Mol Diagn.

[45] Lollo B, Steele F, Gold L (2014). Beyond antibodies: new affinity reagents to unlock the proteome. Proteomics.

[46] Jenison RD, Gill SC, Pardi A, Polisky B (1994). High-resolution molecular discrimination by RNA. Science.

[47] Gold L, Ayers D, Bertino J, Bock C, Bock A, Brody EN (2010). Aptamer-based multiplexed proteomic technology for biomarker discovery. PLoS One.

[48] Ostroff RM, Bigbee WL, Franklin W, Gold L, Mehan M, Miller YE (2010). Unlocking biomarker discovery: large scale application of aptamer proteomic technology for early detection of lung cancer. PLoS One.

[49] Jung YJ, Katilius E, Ostroff RM, Kim Y, Seok M, Lee S (2017). Development of a protein biomarker panel to detect non-small-cell lung cancer in Korea. Clin Lung Cancer.

[50] Teng IT, Li X, Yadikar HA, Yang Z, Li L, Lyu Y (2018). Identification and characterization of DNA aptamers specific for phosphorylation epitopes of tau protein. J Am Chem Soc.

[51] Lundberg M, Eriksson A, Tran B, Assarsson E, Fredriksson S (2011). Homogeneous antibody-based proximity extension assays provide sensitive and specific detection of low-abundant proteins in human blood. Nucleic Acids Res.

[52] Assarsson E, Lundberg M, Holmquist G, Bjorkesten J, Thorsen SB, Ekman D (2014). Homogenous 96-plex PEA immunoassay exhibiting high sensitivity, specificity, and excellent scalability. PLoS One.

[53] Thorsen SB, Lundberg M, Villablanca A, Christensen SL, Belling KC, Nielsen BS (2013). Detection of serological biomarkers by proximity extension assay for detection of colorectal neoplasias in symptomatic individuals. J Transl Med.

[54] Eltahir M, Isaksson J, Mattsson JSM, Karre K, Botling J, Lord M (2021). Plasma proteomic analysis in non-small cell lung cancer patients treated with PD-1/PD-L1 blockade. Cancers (Basel).

[55] Berggrund M, Enroth S, Lundberg M, Assarsson E, Stalberg K, Lindquist D (2019). Identification of candidate plasma protein biomarkers for cervical cancer using the multiplex proximity extension assay. Mol Cell Proteomics.

[56] Enroth S, Berggrund M, Lycke M, Lundberg M, Assarsson E, Olovsson M (2018). A two-step strategy for identification of plasma protein biomarkers for endometrial and ovarian cancer. Clin Proteomics.

[57] Enblad G, Karlsson H, Gammelgard G, Wenthe J, Lovgren T, Amini RM (2018). A phase I/IIa trial using CD19-targeted third-generation CAR T cells for lymphoma and leukemia. Clin Cancer Res.

[58] Liu S, Shen M, Hsu EC, Zhang CA, Garcia-Marques F, Nolley R (2021). Discovery of PTN as a serum-based biomarker of pro-metastatic prostate cancer. Br J Cancer.

[59] Petrera A, von Toerne C, Behler J, Huth C, Thorand B, Hilgendorff A (2021). Multiplatform approach for plasma proteomics: complementarity of Olink proximity extension assay technology to mass spectrometry-based protein profiling. J Proteome Res.

[60] Enroth S, Berggrund M, Lycke M, Broberg J, Lundberg M, Assarsson E (2019). High throughput proteomics identifies a high-accuracy 11 plasma protein biomarker signature for ovarian cancer. Commun Biol.

[61] Ooi AT, Ruff DW (2019). Simultaneous targeted detection of proteins and RNAs in single cells. Methods Mol Biol.

[62] Reimegård J, Tarbier M, Danielsson M, Schuster J, Baskaran S, Panagiotou S (2021). A combined approach for single-cell mRNA and intracellular protein expression analysis. Commun Biol.

[63] Paweletz CP, Charboneau L, Bichsel VE, Simone NL, Chen T, Gillespie JW (2001). Reverse phase protein microarrays which capture disease progression show activation of pro-survival pathways at the cancer invasion front. Oncogene.

[64] Akbani R, Becker KF, Carragher N, Goldstein T, de Koning L, Korf U (2014). Realizing the promise of reverse phase protein arrays for clinical, translational, and basic research: a workshop report: the RPPA (Reverse Phase Protein Array) society. Mol Cell Proteomics.

[65] Tibes R, Qiu Y, Lu Y, Hennessy B, Andreeff M, Mills GB (2006). Reverse phase protein array: validation of a novel proteomic technology and utility for analysis of primary leukemia specimens and hematopoietic stem cells. Mol Cancer Ther.

[66] Petricoin E, Wulfkuhle J, Howard M, Pierobon M, Espina V, Luchini A (2019). RPPA: origins, transition to a validated clinical research tool, and next generations of the technology. Adv Exp Med Biol.

[67] Partolina M, Thoms HC, MacLeod KG, Rodriguez-Blanco G, Clarke MN, Venkatasubramani AV (2017). Global histone modification fingerprinting in human cells using epigenetic reverse phase protein array. Cell Death Discov.

[68] Wang J, Zhao W, Guo H, Fang Y, Stockman SE, Bai S (2018). AKT isoform-specific expression and activation across cancer lineages. BMC Cancer.

[69] Li J, Akbani R, Zhao W, Lu Y, Weinstein JN, Mills GB (2017). Explore, visualize, and analyze functional cancer proteomic data using the cancer proteome atlas. Cancer Res.

[70] Hoff FW, Hu CW, Qutub AA, de Bont E, Horton TM, Kornblau SM (2018). Shining a light on cell signaling in leukemia through proteomics: relevance for the clinic. Expert Rev Proteomics.

[71] Hellstrom C, Dodig-Crnkovic T, Hong MG, Schwenk JM, Nilsson P, Sjoberg R (2017). High-density serum/plasma reverse phase protein arrays. Methods Mol Biol.

[72] Grote T, Siwak DR, Fritsche HA, Joy C, Mills GB, Simeone D (2008). Validation of reverse phase protein array for practical screening of potential biomarkers in serum and plasma: accurate detection of CA19-9 levels in pancreatic cancer. Proteomics.

[73] Yanagita K, Nagashio R, Jiang SX, Kuchitsu Y, Hachimura K, Ichinoe M (2018). Cytoskeleton-associated protein 4 is a novel serodiagnostic marker for lung cancer. Am J Pathol.

[74] Kobayashi M, Nagashio R, Jiang SX, Saito K, Tsuchiya B, Ryuge S (2015). Calnexin is a novel sero-diagnostic marker for lung cancer. Lung Cancer.

[75] Valencia K, Montuenga LM (2021). Exosomes in liquid biopsy: the Nanometric world in the pursuit of precision oncology. Cancers.

[76] Vinik Y, Ortega FG, Mills GB, Lu Y, Jurkowicz M, Halperin S (2020). Proteomic analysis of circulating extracellular vesicles identifies potential markers of breast cancer progression, recurrence, and response. Sci Adv.

[77] Signore M, Alfonsi R, Federici G, Nanni S, Addario A, Bertuccini L (2021). Diagnostic and prognostic potential of the proteomic profiling of serum-derived extracellular vesicles in prostate cancer. Cell Death Dis.

[78] Bhardwaj M, Gies A, Weigl K, Tikk K, Benner A, Schrotz-King P (2019). Evaluation and validation of plasma proteins using two different protein detection methods for early detection of colorectal cancer. Cancers (Basel).

[79] Finkernagel F, Reinartz S, Schuldner M, Malz A, Jansen JM, Wagner U (2019). Dual-platform affinity proteomics identifies links between the recurrence of ovarian carcinoma and proteins released into the tumor microenvironment. Theranostics.

[80] Rossi G, Mu Z, Rademaker AW, Austin LK, Strickland KS, Costa RLB (2018). Cell-free DNA and circulating tumor cells: comprehensive liquid biopsy analysis in advanced breast cancer. Clin Cancer Res.

[81] Brunner A-D, Thielert M, Vasilopoulou CG, Ammar C, Coscia F, Mund A, et al. Ultra-high sensitivity mass spectrometry quantifies single-cell proteome changes upon perturbation. bioRxiv. 2021:2020.12.22.423933. 10.15252/msb.202110798 PMC8884154 35226415

[82] Stoeckius M, Hafemeister C, Stephenson W, Houck-Loomis B, Chattopadhyay PK, Swerdlow H (2017). Simultaneous epitope and transcriptome measurement in single cells. Nat Methods.

[83] Chen Z, Chen JJ, Fan R (2019). Single-cell protein secretion detection and profiling. Annu Rev Anal Chem (Palo Alto, Calif).

